# We like to move it, move it: A perspective on performing passive leg movement as a non-invasive assessment of vascular function in pediatric populations

**DOI:** 10.3389/fphys.2023.1165800

**Published:** 2023-04-26

**Authors:** Michele N. D’Agata, Alexs A. Matias, Melissa A. Witman

**Affiliations:** Vascular Function in Chronic Disease Research Laboratory, Department of Kinesiology and Applied Physiology, University of Delaware, Newark, DE, United States

**Keywords:** passive leg movement, feasibility, children, adolescents, age, vascular function

## Abstract

The passive leg movement (PLM) technique is a non-invasive assessment of lower-limb vascular function. PLM is methodologically simple to perform and utilizes Doppler ultrasound to determine leg blood flow (LBF) through the common femoral artery at rest and in response to passive movement of the lower leg. LBF responses to PLM have been reported to be mostly nitric oxide (NO)-mediated when performed in young adults. Moreover, PLM-induced LBF responses, as well as the NO contribution to PLM-induced LBF responses, are reduced with age and in various diseased populations, demonstrating the clinical utility of this non-invasive test. However, no PLM studies to date have included children or adolescents. Since its conception in 2015, our laboratory has performed PLM on hundreds of individuals including a large cohort of children and adolescents. Thus, the purpose of this perspective article is threefold: 1) to uniquely discuss the feasibility of performing PLM in children and adolescents, 2) to report PLM-induced LBF values from our laboratory in 7–17-year-olds, and 3) to discuss considerations for making comparisons among pediatric populations. Based on our experiences performing PLM in children and adolescents (among various other age groups), it is our perspective that PLM can feasibly be performed in this population. Further, data from our laboratory may be used to provide context for typical PLM-induced LBF values that could be observed in children and adolescents, as well as across the lifespan.

## Introduction

The passive leg movement (PLM) technique is a reliable, non-invasive assessment of lower limb vascular function (Gifford and Richardson, 2017; Lew, Liu et al., 2021; Groot, Broxterman et al., 2022). PLM requires the use of Doppler ultrasound to evaluate common femoral artery (CFA) diameter and blood velocity at rest and during passive movement of the lower leg at the knee joint through a 180-90-180-degree range of motion (Gifford and Richardson, 2017). At its essence, PLM is methodologically simple to perform, though the mechanisms of the test are a bit more complex. In brief, the results of PLM are mainly interpreted as PLM-induced changes in leg blood flow (LBF) compared to baseline LBF (Gifford and Richardson, 2017). The mechanisms driving these PLM-induced increases in LBF begin with the initiation of the movement phase of the test, resulting in mechanical deformation of muscle fibers in the moved leg. Passive muscle stretch induces positive changes in both central and peripheral hemodynamics via activation of the mechanoreflex and endothelial cell release of nitric oxide (NO), respectively (Jufri, Mohamedali et al., 2015; Kruse, Hughes et al., 2018). The increase in central hemodynamics and peripheral vasodilation simultaneously increase perfusion pressure and shear stress to drive transient, yet robust changes in LBF (Trinity, Amann et al., 2010). Importantly, LBF responses to PLM have been documented to be 80%–90% NO mediated when performed in healthy young adult men (Mortensen, Askew et al., 2012; Trinity, Groot et al., 2012). Given that reduced NO bioavailability is a hallmark of vascular endothelial dysfunction leading to the development of cardiovascular disease (Cannon, 1998), PLM presents as a useful and insightful tool for evaluating peripheral vascular function.

Accordingly, PLM has been employed in a variety of populations in both health and disease. With healthy human aging, cross sectional studies demonstrate that LBF responses to PLM are significantly attenuated in old versus young adults (Groot, Rossman et al., 2015; Trinity, Groot et al., 2015). Similarly, sedentary older adults have been found to have reduced PLM-induced LBF responses compared to physically active older adults (Groot, Rossman et al., 2016). PLM has also provided insight into the effects of numerous disease states on central hemodynamics and peripheral microvascular function including heart failure, heart transplantation, peripheral artery disease, chronic obstructive pulmonary disease, chronic kidney disease, systemic sclerosis, spinal cord injury, and sepsis (Hayman, Nativi et al., 2010; Mortensen, Askew et al., 2012; Venturelli, Amann et al., 2014; Witman, Ives et al., 2015; Nelson, Rossman et al., 2016; Clifton, Machin et al., 2018; Katulka, Hirt et al., 2019; Ives, Layec et al., 2020). As expected, in each of these studies we see that disease has a negative impact on at least one component of the PLM response (central or peripheral) as compared to healthy controls.

Our laboratory has more recently utilized PLM to assess the impact of the menstrual cycle (D'Agata, Hoopes et al., 2021) and sleep regularity (Hoopes, Berube et al., 2021) on peripheral vascular function in healthy young adults. To date, our employment of the PLM technique has provided us with novel insights into how disease, sex hormone concentrations, and sleep patterns influence the peripheral vasculature. Collectively, findings to date implicate PLM as a useful, insightful, and methodologically simple assessment of peripheral microvascular function that is predominantly NO mediated and can be used in a variety of populations. However, a notable gap in the literature is that PLM has not yet been reported in pediatric populations.

### PLM & alternative vascular function techniques

When assessing vascular function in all individuals, but particularly for pediatric populations, the ideal assessment would be non-invasive, short in duration, free of general discomfort to the participant, and consist of methodology that can be easily understood by the child, clinician, and/or researcher. PLM represents a vascular function test that meets most, if not all of these criteria. Methodologically, a trained sonographer locates the CFA and maintains a clear image and blood velocity tracing for a 1-min baseline period, and during 2 min of continuous passive leg movement. The PLM technique is performed by an assistant passively moving the participant’s lower leg at the knee joint in a 180-90-180-degree range of motion at a frequency of 1-hertz, while being cognizant of any resistance to passive movement by the participant (Gifford and Richardson, 2017). Due to this straightforward methodology, PLM is simple to describe to non-experts and has been easily understood by pediatric participants in our laboratory. Additionally, PLM is non-invasive with active data collection only lasting 3–4 min in duration, (Gifford and Richardson, 2017), and anecdotally, our pediatric cohort has not reported any pain or discomfort with the technique.

In contrast, non-invasive vascular tests that have been traditionally performed in pediatric populations include brachial or femoral artery flow-mediated dilation (FMD), peripheral arterial tonometry (PAT), and venous occlusion strain gauge plethysmography (VOP), with brachial artery FMD being most often utilized (Sorensen, Celermajer et al., 1994; Duck and Hoffman, 2007; Bruyndonckx, Radtke et al., 2013). Although each of these tests have their strengths and value, there are several considerations that make PLM an attractive test for assessing vascular function in pediatric populations. As compared to each of the other non-invasive assessments, active data collection for PLM is the shortest in duration at ∼3–4 min (Gifford and Richardson, 2017) while FMD is between ∼8 and 10 min (Celermajer, Sorensen et al., 1992; Sorensen, Celermajer et al., 1994; Harris, Nishiyama et al., 2010), PAT is ∼15 min (Bruyndonckx, Radtke et al., 2013), and VOP is ∼8 min (Duck and Hoffman, 2007). Similarly, the recovery time for within-day repeated measurements is relatively short for PLM at ∼5 min, though we recommend a recovery time closer to 10 min. Comparatively, PAT and brachial FMD both recommend ≥30 min of recovery time between repeat assessments (Harris, Padilla et al., 2006; Wood and Stewart, 2010; Vizzardi, Gavazzoni et al., 2014). Finally, PLM is least likely to cause discomfort to the participant given that it is the only test that does not employ constrictive occlusion cuffs to alter blood flow, which often lead to discomfort at, and distal to, the occlusion site. Indeed (Van De Maele, Devlieger et al., 2021), reported that ∼40% of 4–11-year old’s experienced some level of pain following PAT. Constrictive occlusion likely contributed to the sensation of pain reported by the participants, and although minimal, it still underscores a major limitation of cuff occlusion-based vascular techniques particularly in children.

### Feasibility of performing PLM in youth in our laboratory

In our lab, we have attempted PLM on 73 typically developing children and adolescents between the ages of 7–17 years old, which resulted in 66 successful vascular assessments yielding a 90% success rate. Our PLM success rate is similar, if not slightly better, than that reported for alternative vascular functions tests including brachial FMD [87% success rate in typically developing 9–11-year-olds (Leeson, Whincup et al., 1997)] and fingertip PAT [83.1% rate in typically developing 6–11-year-olds (Van De Maele, Devlieger et al., 2021)]. Moreover, familiarizing participants to PLM has shown to improve test quality, and PLM can be completed quickly with both minimal error and participant discomfort. Furthermore, all parents/guardians consented to testing and none reported concerns with performing PLM on their child. Instead, the limiting factor in the feasibility of performing PLM in participants of any age is their ability to remain passive during movement. In our lab, of the 7 unsuccessful attempts, 6 were due to the inability of the participant to remain passive and 1 was due to participant refusal to undergo testing. We have observed comparable success rates in our adult cohorts, suggesting similar feasibility among pediatrics and adults. In our experience, familiarizing the participant to PLM and coaching the participant to remain passive can largely help to successfully achieve passive movement. Still, a few individuals (children and adults) may be unable to remain passive, a limitation inherent to the nature of PLM.

### PLM-induced LBF values from our laboratory

**FIGURE 1:**
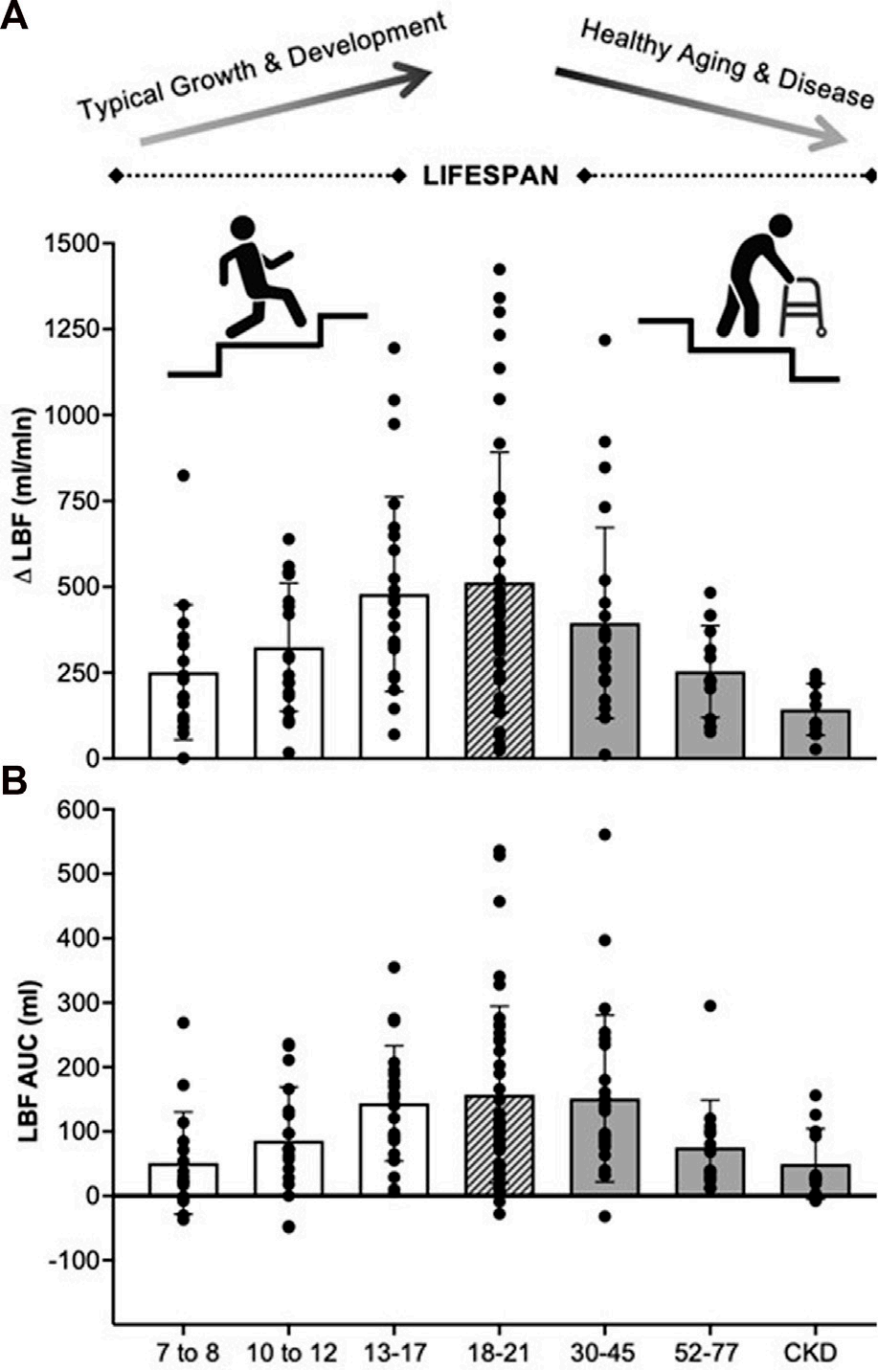
Absolute change in leg blood flow **(A)** and leg blood flow area under the curve **(B)** responses to PLM for various age and health status groups recently collected in our laboratory. 7–8 years (*n* = 16, M/F: 9/7), 10–12 years (*n* = 19, M/F: 11/8), 13–17 years (*n* = 24, M/F: 12/12), 18–21 years (*n* = 40, M/F: 14/26), 30–45 years (*n* = 24, M/F: 8/16), 52–77 years (*n* = 14, M/F: 5/9), CKD (*n* = 12, M/F: 7/5). For illustrative purposes only. LBF, leg blood flow; Δ, absolute change; AUC, area under the curve, CKD, chronic kidney disease.

We have demonstrated that PLM is a methodologically sound tool to assess lower-limb microvascular function in typically developing pediatrics. Table 1 reports responses to PLM as non-normalized and normalized [to body surface area (BSA) (Du Bois and Du Bois, 1916)] values, as well as participant characteristics, obtained from our laboratory for three pediatric age groups (7-8-, 10–12-, and 13–17-year olds). We see a pattern of change with all non-normalized PLM variables across our three cohorts: namely, a stepwise increase with age in baseline CFA diameter, baseline LBF, peak LBF, the absolute change in LBF (ΔLBF), and LBF area under the curve (AUC). Figure 1 visually demonstrates these increases in non-normalized ΔLBF and LBF AUC as bar graphs. Notably, we also observe a concurrent increase in anthropometrics including height, weight, body mass index, and BSA (Table 1), which may be of important consideration when interpreting PLM values in pediatric populations.

**TABLE 1:** Participant characteristics and PLM values in distinct pediatric age groups.

Participant characteristics	7–8 years old (*n* = 16, M/F: 9/7)	10–12 years old (*n* = 19, M/F: 11/8)	13–17 years old (*n* = 24, M/F: 12/12)
Height (cm)	133 ± 7	152 ± 9	166 ± 10
Weight (kg)	31 ± 6	45 ± 11	60 ± 11
BMI (kg/m^2^)	18 ± 2	20 ± 4	21 ± 3
BSA (m^2^)	1.07 ± 0.13	1.38 ± 0.18	1.66 ± 0.18
PLM Values (Non-Normalized)			
Baseline LBF (mL/min)	206 ± 81	249 ± 120	273 ± 105
Peak LBF (mL/min)	457 ± 201	573 ± 212	752 ± 331
ΔLBF (mL/min)	252 ± 197	324 ± 187	479 ± 283
LBF AUC (mL)	51 ± 79	86 ± 83	144 ± 89
PLM Values (Normalized to BSA)			
Baseline LBF (mL/min/m^2^)	192 ± 77	179 ± 77	164 ± 61
Peak LBF (mL/min/m^2^)	423 ± 177	416 ± 145	450 ± 177
ΔLBF (mL/min/m^2^)	231 ± 180	237 ± 139	285 ± 152
LBF AUC (mL/m^2^)	48 ± 75	65 ± 65	86 ± 51
CFA Diameters (mm)			
Baseline	0.58 ± 0.06	0.68 ± 0.10	0.75 ± 0.08
Movement diameter*	0.57 ± 0.08	0.66 ± 0.10	0.75 ± 0.08

BMI, body mass index; BSA, body surface area determined by the Du Bois equation; LBF, leg blood flow; Δ, absolute change; AUC, area under the curve; CFA, common femoral artery; PLM, passive leg movement. *CFA diameter was retrospectively measured during the movement at seconds 12, 24, 36, 48, and 60 for participants with high image quality (*n* = 51); Movement diameter was then calculated as the average of the FA diameters measured ruing movement.

Indeed, a key feature of pediatric development is a gradual and consistent increase in anthropometrics. The increase in weight observed in typically developing children is largely due to an increase in lean muscle mass (Rogol, Roemmich et al., 2002). Moreover, vascular remodeling during longitudinal growth may influence the degree of mechanical deformation driving flow-induced dilation, i.e., the peripheral contribution to hyperemia (Clifford, Kluess et al., 2006; Kirby, Carlson et al., 2007). This is important as PLM-induced hyperemia is partly driven by muscle volume-dependent factors, like NO (Clifford, Kluess et al., 2006; Kirby, Carlson et al., 2007). Furthermore, in denervated and atrophied mice, changes in muscle mass precede structural changes in the vasculature, underscoring the tight relations between skeletal muscle and the vasculature (Ichinose, Kurose et al., 2008). It is therefore reasonable to assume that the magnitude of PLM-induced hyperemia in pediatrics is contingent upon muscle volume.

In an attempt to indirectly explore the role of muscle volume, PLM-induced hyperemia values were normalized to BSA—a surrogate index of muscle volume. Normalizing LBF responses greatly reduced the magnitude of the stepwise increase with age but did not completely abolish all observed differences (Table 1). However, it is important to recognize that BSA does not capture lean muscle mass with precision and is thus not a direct substitute to lean muscle volume. Therefore, the pediatric utility of PLM may greatly benefit from a complimentary assessment of lower limb muscle volume, which we know changes with normal growth and development. Accounting for muscle volume may be especially important when following subjects across time (e.g., childhood into adolescence), or comparing pediatric populations with large variations in muscle volume (e.g., overweight/obese). Fortunately, feasible caliper-based methods of assessing leg muscle volume have been developed and validated (Jones and Pearson, 1969; Andersen and Saltin, 1985) with gold standard ^1^H-Magnetic Resonance Imaging, which we have recently begun to employ in our laboratory (Layec, Venturelli et al., 2014).

Lastly, in Figure 1 we also present non-normalized LBF data from our lab for healthy young (18–21 years), middle-aged (30–45 years), and older (52–77 years) adults, as well as patients with chronic kidney disease (CKD), to provide context for the changes across the lifespan. Here we observe gradual decrements in ΔLBF and LBF AUC with increasing age from young to older adults, and in disease, suggestive of declining vascular function. Importantly, these findings replicate the literature, demonstrating impairments in hyperemia to PLM with increasing age (Groot, Rossman et al., 2015; Trinity, Groot et al., 2015) and in disease states known to negatively impact vascular health (Hayman, Nativi et al., 2010; Mortensen, Askew et al., 2012; Venturelli, Amann et al., 2014; Witman, Ives et al., 2015; Nelson, Rossman et al., 2016; Clifton, Machin et al., 2018; Ives, Layec et al., 2020). In contrast to declining vascular function with healthy aging and disease, the lower observed hyperemic responses in our 7-8- and 10–12-year olds is likely a function of factors innate to the pediatric life stage. Beyond the previously discussed differences in muscle volume, other factors such as developmentally appropriate increases in blood pressure could also be driving the observed increases in hyperemia from childhood to adolescence.

### Additional methodological considerations

Understanding the impact of PLM on CFA diameter in children and adolescents is an important methodological consideration when interpreting movement-induced hyperemia. In adults, CFA diameter does not dilate during passive movement of the lower leg (Gifford and Richardson, 2017). Instead, the hyperemic response to PLM is largely determined by dilation of resistance vessels distal to the CFA, i.e., downstream microvascular function. However, it was unknown if the CFA dilates during PLM in typically developing pediatrics. We therefore collected measurements of CFA diameter during the movement phase of PLM at 5 distinct time points (12, 24, 36, 48, and 60 s) on CFA scans with high image quality (n = 51). Average baseline and movement diameters for each pediatric age group are displayed in Table 1. There was no change in CFA over time from baseline throughout movement for the whole group [baseline: 0.69 ± 0.10; movement: 0.68 ± 0.12, 0.68 ± 0.12, 0.67 ± 0.12, 0.68 ± 0.11, 0.67 ± 0.11 cm (one-way repeated measures ANOVA: *p* = 0.36)]. Importantly, these data indicate that PLM does not alter CFA diameter in a group of children and adolescents supporting its assessment of primarily microvascular function in this population.

Neurohumoral changes associated with sexual maturation, such as increases in sex hormone concentrations, likely contribute to the balance of NO and NO-independent factors, as well as central and peripheral responses to hyperemia (Ahimastos, Formosa et al., 2003). Our lab and other groups have observed sex-specific differences in the central hemodynamic response to PLM (Ives, McDaniel et al., 2013) as well as the relatedness of PLM to brachial artery flow-mediated dilation (Rossman, Groot et al., 2016; D'Agata, Hoopes et al., 2022) in healthy young adults. As such, the potential for sex differences is an important consideration when interpreting movement-induced hyperemia and requires better understanding to enhance implementation of PLM in pediatrics, particularly during and after the pubertal transition. Further, there is currently much debate about the role of sex hormones on vascular function, particularly throughout the menstrual cycle (Wenner and Stachenfeld, 2020). Although complete discussion of this topic is beyond the scope of this perspective, in the data presented we did purposefully control for the menstrual cycle by testing our female adolescents during the early follicular phase (i.e., days 1–7 following onset of menstruation) when applicable.

### Future directions

As previously mentioned, LBF responses to PLM have been documented to be ∼80–90% NO mediated (Mortensen, Askew et al., 2012; Trinity, Groot et al., 2012), corroborating this technique as an index of microvascular endothelial NO bioavailability. However, these initial studies were only conducted in healthy young adult men, limiting generalizability. Determining the mechanisms of PLM in typically developing pediatric populations would greatly aid in our understanding and interpretation of PLM as a vascular assessment. However, we must be conscious of the ethical constrains to employing direct, invasive mechanistic studies in this population (e.g., NO-blockade). Therefore, indirect mechanistic studies, such as venous blood samples to evaluate NO metabolites is a potential area for future research. Additionally, real-time BP measurements are needed to investigate leg vascular conductance (LVC) in this population. Parsing out the role of central hemodynamics in typically developing pediatrics would thus allow for better understanding of LBF responses across the lifespan.

Our laboratory has demonstrated the feasibility of performing PLM, but whether it is reproducible in a pediatric cohort remains to be explored. To quote Atkinson & Nevil, 1998: *“…it is reliability that should be tested for first in a new measurement tool, since it will never be valid if it is not adequately consistent in whatever value it indicates from repeated measurements.”* Studies on the absolute and relative reproducibility of PLM in pediatric populations are thus warranted. Such information would allow clinicians and researchers to monitor changes in vascular health and/or regulation throughout pubertal development (absolute), as well as detect and measure systematic differences between groups (relative). The application of PLM would therefore benefit from its ability to monitor LBF patterns across time, bolstering the diagnostic and prognostic utility of this technique. Validity studies on PLM are also important if movement-induced hyperemia is to be used as a complementary clinical assessment in pediatrics. Following confirmation of reproducibility and validity in typically developing pediatrics, future research regarding the use of PLM in special pediatric populations will be needed to better understand the clinical utility of this vascular technique in these younger cohorts. For example, the alarming rise in childhood obesity and sedentarism (Lakshman, Elks et al., 2012) has propagated the development of other childhood cardiometabolic diseases including type II diabetes, hypertension, and dyslipidemia (Chung, Onuzuruike et al., 2018). It is well known that vascular dysfunction precedes overt cardiovascular diseases, and thus, may serve as a preclinical index of cardiovascular health (Anderson, Uehata et al., 1995). Opportunely, PLM presents a promising tool to assess early changes in vascular function in diseased pediatric populations at increased risk of cardiometabolic morbidity. Furthermore, application of PLM may be especially encouraging in clinical settings where concerns regarding frailty and discomfort (e.g., muscular dystrophy) have deterred the use of more invasive assessments of vascular function.

## Conclusion

It is our perspective that PLM is a feasible tool to assess peripheral microvascular function in typically developing children and adolescents. Findings to date implicate PLM as useful, insightful, and methodologically simple, lending credence to this vascular technique as a promising diagnostic and prognostic tool. Although evidence corroborates PLM as an assessment of NO-mediated microvascular function in adults, additional less invasive studies are warranted to verify if this NO dependency translates to pediatrics. Furthermore, a better appreciation of the role of sex and pubertal development on movement-induced hyperemia is needed. Nonetheless, the PLM technique provides a unique window into vascular function during childhood and adolescence.

## Data Availability

The raw data supporting the conclusion of this article will be made available by the authors, without undue reservation.
